# Oil tanker under ice loadings

**DOI:** 10.1038/s41598-023-34606-w

**Published:** 2023-05-29

**Authors:** Oleg Gaidai, Ping Yan, Yihan Xing, Jingxiang Xu, Fuxi Zhang, Yu Wu

**Affiliations:** 1 Shanghai Ocean University, Shanghai, China; 2 University of Stavanger, Stavanger, Norway

**Keywords:** Environmental sciences, Ocean sciences, Engineering, Mathematics and computing

## Abstract

As a result of global warming, the area of the polar pack ice is diminishing, making merchant travel more practical. Even if Arctic ice thickness reduced in the summer, fractured ice is still presenting operational risks to the future navigation. The intricate process of ship-ice interaction includes stochastic ice loading on the vessel hull. In order to properly construct a vessel, the severe bow forces that arise must be accurately anticipated using statistical extrapolation techniques. This study examines the severe bow forces that an oil tanker encounters when sailing in the Arctic Ocean. Two stages are taken in the analysis. Then, using the FEM program ANSYS/LS-DYNA, the oil tanker bow force distribution is estimated. Second, in order to estimate the bow force levels connected with extended return periods, the average conditional exceedance rate approach is used to anticipate severe bow forces. The vessel’s itinerary was planned to take advantage of the weaker ice. As a result, the Arctic Ocean passage took a meandering route rather than a linear one. As a result, the ship route data that was investigated was inaccurate with regard to the ice thickness data encountered by a vessel yet skewed with regard to the ice thickness distribution in the region. This research intends to demonstrate the effective application of an exact reliability approach to an oil tanker with severe bow forces on a particular route.

## Introduction

Oil tanker designs must be secure, sturdy, and reliable due to an increase in marine operations in the Arctic connected to oil and gas exploration and transportation^1,2^. There is a need for a comprehensive research of the associated severe force statistical distribution operating on the oil tanker bow area because ship-ice interaction is a complicated random nonlinear process that depends on in situ ice thickness distribution^1,3,4^.

Data on onboard measured ice thickness are seldom available in the scientific literature at this time^3^, and only limited experimental measurements^5^. It is crucial to take into account the valid range of ice thickness and its probability distribution, route-specific and vessel-specific, when calculating oil tanker bow forces. This study makes use of actual ice thickness measurements taken on board an icebreaker traveling along the 90's and 60's meridian systems towards the direction of the North Pole and back^6,7^. By photographing cracked ice pieces, the thickness of the ice was measured.

The number of ships operating in the arctic area has constantly expanded as a result of the exploitation of polar resources and the creation of Arctic shipping routes. In the research of ship-ice collision, broken ice should be taken into consideration as usual polar ice. The techniques for creating shattered ice aren't finished yet, though. Now, a place for cracked ice is created using the notion of cellular automata. The Voronoi diagram utilizes the cell points to create a polygonal simulation model of shattered ice. With the use of Mean Calliper Diameter (MCD) theory, the shattered ice simulation model's veracity may be confirmed. The thickness, probability distribution, and scale for shattered ice are then individually optimized. To make the broken ice model as near to the real broken ice situation as possible, the optimized value will be optimized after each phase of optimization and verified using MCD theory. For the purpose of building the polar ice-breaking model, it might offer a precise reference meaning.

This study was motivated by growing industrial interest to Arctic Ocean potential navigation areas. Authors therefore intend to contribute to the safe and reliable design of large vessels suitable for Arctic Ocean oil tanker operations. Table 1 presents oil tanker selected specifications.

**Table 1:** Selected oil tanker specifications.

Type	Overall length (L_OA_)	Waterline length (L_WL_)	Length between perpendiculars (L_PP_)	Moulded breadth (B)
Oil tanker	150.6 m	146.0 m	143.0 m	20.5 m
Moulded depth (D)	Designed draft(d)	Beam camber	Speed	Drainage volume
11.2 m	8.4 m	0.35 m	14.6 kn	18.2 kt
Capacity of cargo holds	Host model	Rated power	Rated speed	Classification of vessel
15,400 m^3^	8PC2-6L	4400 KW	520 rpm	CCS

The oil tanker vessel used in this study is designated as Polar Class 4 (PC4), and it operates under yearly heavy ice conditions, including old ice, for example between 1 and 2 m thick. The relevance of vessel operations and transit under ice effect is growing as the Arctic is being explored more. Extreme bow force estimation for oil tankers is a crucial component of the preliminary design and has a significant impact on the vessel's overall performance while in operation. Although the ultimate limit states (ULS) are often based on precise prediction of extreme loads, the extreme values of oil tanker bow loads (and therefore huge bow forces) are closely related to the reliability of the whole vessel^8^. Most extreme ice load research studies utilize classic extreme value distributions (EVD) theory^1,9^. As for engineering purposes, ice loads, estimated only from expected ice thickness distribution, but without accounting for the actual ship hull geometry, are insufficient, thus the primary target should be oil tanker bow geometry-specific force pattern. This study's primary contribution is applying modified Weibull method^10–23^, to the oil tanker route-specific ice thickness data with the goal of estimating the crucial stresses at the oil tanker bow.

Apart from route-specific ice-thickness, sea ice concentration being an important factor for potential commercial navigation through Arctic Polar Regions. A 25 by 25 km grid of sea ice concentrations for Arctic Polar Regions are provided by the Sea Ice Concentration Climate Data Record (CDR). This information may be used to calculate the percentage of the ocean's surface that is covered by ice and to track variations in the concentration of sea ice. For an oil tankers vessel to operate dependably and be safer to build, proper reliability approach is required. The results of this work may be classified into two categories: statistical technique and ice collision modelling. Although there is a lot of current relevant research in the field of ice collision modelling^1,24–32^. The authors of this paper wanted to focus attention on the vital subject of oil tanker transportation in the Arctic. The originality of this study comes in its attempt to examine the reliability of oil tankers during upcoming Arctic voyages; as a result, the topic posed on its own is pertinent to naval architecture in the very near future. The reliability technique used in this study is extremely general and may be easily adapted to other buildings and environmental conditions that are similar^2–4,6,33–35^.

### Ice thickness distribution

**Figure 1:**
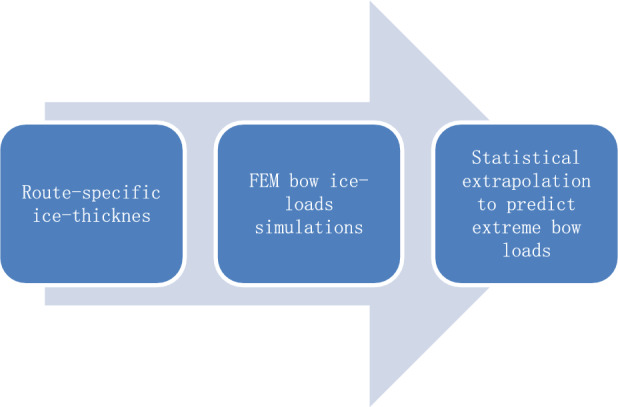
Suggested methodology flow chart.

The icebreaker route follows a meandering rather than a straight line. This study is skewed since it uses statistical data on ice thickness that was observed along a particular Arctic route. The prejudice was brought on by particular route selections and particular seasons. Notice that the vessel carefully avoided regions of heavy, multi-year ice by taking use of extensive polynyas and orienting meridionally. Mean ice thickness was route-specific. Figure 1 sketches methodology, advocated in this study.

As compared to the ice thickness distribution that is peculiar to a certain location, the ice thickness distribution tail (very thick ice) is often underrepresented. The latter is a result of avoiding the areas with heavier ice. All measurements of vessel ice thickness have this bias built-in. The unbiased distribution of ice thickness is known as “region-specific”, whereas this biased distribution is often known as “route-specific”^6,7^. In the probabilistic study of ice-induced loads on oil tankers, the ice thickness distribution reflects the ice that the vessel will actually encounter. As the distribution of ice thickness is inherently constrained on both sides, the so-called Beta probability distribution function (PDF) may be used to approximate it

1 $$p\left(h\right)=K{h}^{\beta -1}{\left(1-h\right)}^{\alpha -1}$$

with $K=\frac{\Gamma \left(\alpha +\beta \right)}{\Gamma \left(\alpha \right)\Gamma \left(\beta \right)}$, and $p\left(h\right)$ being PDF for non-dimensional ice thickness $h=H/{H}_{\text{max}}$, with $H$ being encountered ice thickness (in meters), and ${H}_{\text{max}}$ being the assumed maximum (cut-off) encountered ice thickness, and $\Gamma$ being the Gamma function. Apart from Beta distribution, given by Eq. (1), other more advanced distributions may be well considered^6^; to mention few: generalised Gamma distribution, two-parametric Weibull distribution^6,7^, log-normal distribution^36^ – Note that some are less suited since they lack an upper cut-off limit. This study's objective was to effectively use the raw sampling empirical distribution with the following practical calculation of critical bow forces rather than to determine which ice thickness distribution matches best. The beta-distribution, which has the benefit of having bounded support, was thus chosen here as an example (cut-off value ${H}_{\text{max}}$).

PDF corresponding to presents a route-specific ice thickness, encountered by actual icebreaker, operating North of 81°N, was used in this study. Ice thickness PDF was then fitted using Beta distribution. Beta distribution PDF parameters have been fitted, using raw estimated mean along with standard deviation^6,7^. Note that ice thickness PDF was cut off at the right-hand distribution tail side. PDF tail cut-off value was corresponding to the ice thickness, that oil tanker cannot, or will not attempt to navigate through. Studied oil tanker was not intended to operate in ice thicker than 2 m, thus cut-off value has been set equal to ${H}_{\text{max}}=$ 2 m.

### Cellular automata icebreaking model

“Cellular automata” is a dynamic system with discrete time and space that meets the criteria for ice-breaking space random distribution, local interaction, and time causality. It can be used to study how simple local rules evolve into more complex global dynamics^29^,. Lu^27^, presented an expanded field of cellular automata to describe how groups of pedestrians move while walking. Zhao^34^, examined the evacuation of individuals with random and aggregation distribution using the cellular automata random model to mimic people with aggregation behaviour. Li^27^, created the pedestrian simulation model using the cellular automata concept. Cellular automata were utilized in the aforementioned investigations to model the disorderly population structure. It is used to examine ice regions and to build the broken ice model, taking into account its random properties.

**Figure 2:**
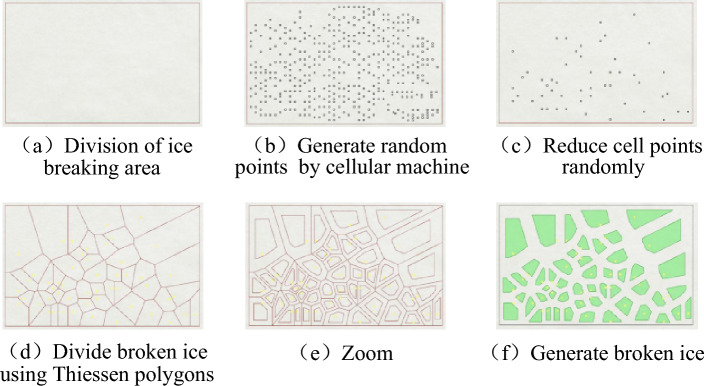
Creating broken ice pattern.

This study used broken ice model implemented by Rhinoceros CAD software^37^, with proper input parameters for geometry and density of broken ice, thus developing ice-breaking creation method in the area close to the actually broken ice zone. MCD (Mean Calliper Diameter) distribution law of broken ice in the actually broken ice area was utilized, based on the theory of cellular automata. The four essential components of cellular automata are the cell, cell space, neighbour, and evolution rules^1,24–34^. Cellular automata exhibit homogeneity, spatial dispersion, and temporal dispersion. As a result of years of intensive study and development, cellular automata currently have important uses in physics, biology, and transportation. It is suggested that cellular automata and ice-breaking architecture be combined to generate the ice-breaking model. Tyson polygons, which may be used to randomly partition 2D polygons and 3D polyhedrons, are a collection of continuous polygons made up of vertical bisection lines linking two neighbouring point segments. The outcomes of this construction procedure are displayed in detail in Fig. 2a–f.

Due to the irregular polygon of broken ice collision that occurs when ships sail in the polar ice region, the size of broken ice should be expressed in a unified manner before establishing the ice model. MCD theory is introduced to represent the size of the broken ice model. MCD is the short form for Mean Caliper Diameter; a specific amount used to express the size of broken ice. MCD is also the equivalent diameter of broken ice. Probability distribution of broken ice MCD and equivalent diameter *D* of broken ice MCD are both taken from^35^, with

2 $$D = l/\pi$$

with *D* being broken ice equivalent diameter (m), and *l* being broken ice circumference (m). The probability distribution of ice fragmentation MCD, based on the actual measured values in the polar ice fragmentation zone, roughly complies with the formula for the negative exponential power function

3 $$f_{MCD} = \frac{ - \beta }{{D_{\max }^{ - \beta } - D_{\min }^{ - \beta } }}D^{ - \beta - 1} ,D \in \left[ {D_{\min } ,D_{\max } } \right]$$

with $\beta$ being variable parameter, determined by the polar ice region geographical location, $D_{\min }$ being broken ice minimum equivalent diameter (m), $D_{\max }$ being broken ice maximum equivalent diameter (m)^35^.

**Figure 3:**
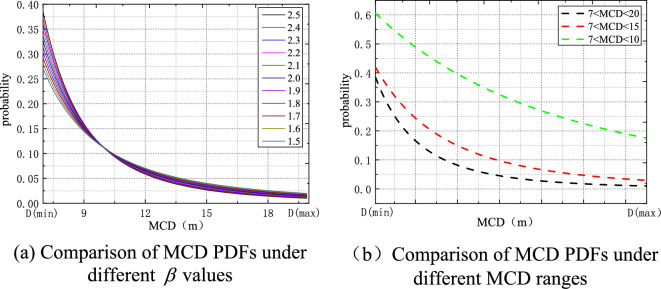
Comparison chart of MCD PDFs.

Using the “sea of Okhotsk (Feb 2003)” measured data as an example, the ice-breaking in the sea region at this time is 7 m. The parameter range was chosen between 1.5 and 2.5 in order to analyse the impact of variable parameters on probability. A probability distribution comparison diagram with various parameters was produced in Fig. 3a. The equivalent diameter *D* of shattered ice serves as the independent variable and is a subtractive function for the MCD probability distribution function presented. Hence, the worth of $D_{\min }$ determines how many broken ice pieces are present in entire broken ice area, whereas the $D_{\max }$ is relatively small in the distribution tail in Fig. 3b.

To find the desired probability function, we settle on a modest value of 1.8. A 40% dense area for cracking ice has been built. It is specifically noted that the equivalent diameter MCD (m) of the ice breaking is too large or too small, which will significantly affect the probability distribution value of ice breaking, due to the influence of the accumulated “difference” of the ice breaking areas under the ice breaking distribution points built by the cellular mechanism. The MCD probability value of the ice-breaking model is closer to the theoretical PDF, especially the significant variance reduction, indicating that the error data dispersion is controlled. The distribution criteria are too regular when creating the broken ice model, which is inconsistent with the random distribution of the broken ice in the real world. The broken ice is evenly scaled based on the Tyson polygon theory so that the distance between the produced fractured ice is identical. The unified scaling factor is optimized to a certain scaling range in order to address this issue. The Tyson polygon method is used to split the fractured ice blocks, and then various scale factors are applied to each broken ice block. The ideal handling of two-dimensional ice breaking and the thickness of ice breaking must be taken into account while creating the ice-breaking model. Broken ice areas of the same thickness are produced using the conventional method of constructing broken ice, which differs from the thickness of broken ice in actual seas. The ice breaking model has been quite near to the actual ice condition after a series of optimizations on the ice breaking scale, probability distribution, and ice breaking thickness. It establishes a strong base for the precision of later numerical simulation. Solid elements with eight free nodes were selected for sea ice model, specific parameters being shown in Table 2.

**Table 2:** Material parameters of sea ice.

Material properties	Density (kg/m^3^)	Shear modulus (GPa)	Yield stress	The bulk modulus (GPa)	Plastic hardening modulus (GPa)	Plastic failure strain
Value	910	2.2	2.1	5.3	4.3	0.35

The parameters of the ice material refer to the relevant compression test research carried out by^38,39^, and other scholars on the ice material. The test data well shows that the isotropic elastoplastic sea ice constitutive model can better describe the compression failure of the sea ice material. In the definition of isotropic elastic–plastic sea ice constitutive model, extrusion strength, plastic failure strain and fracture pressure are considered as the criteria for sea ice failure. The material is an elastic fracture failure model with plastic strain failure criterion. When the effective plastic strain reaches the failure strain or the pressure reaches the failure truncation pressure, the element loses the capacity to bear the stress and the deviatoric stress becomes zero, that is, the material behaves as a fluid state. For more details on failure mechanisms within material ice models see^40–42^. In this study simple LS-DYNA isotropic elastic–plastic material model with failure MAT_ISOTROPIC_ELASTIC_FAILURE was used. Note that focus of this study was reliability method (as indicated in flowchart in Fig. 1), and not numerical/material side of LS-DYNA. In other words, authors presented general purpose reliability approach for potential (in the near future) oil tanker Arctic navigation, and presented numerical and material setup has been chosen as illustrative example.

### Bow force assessment

**Figure 4:**
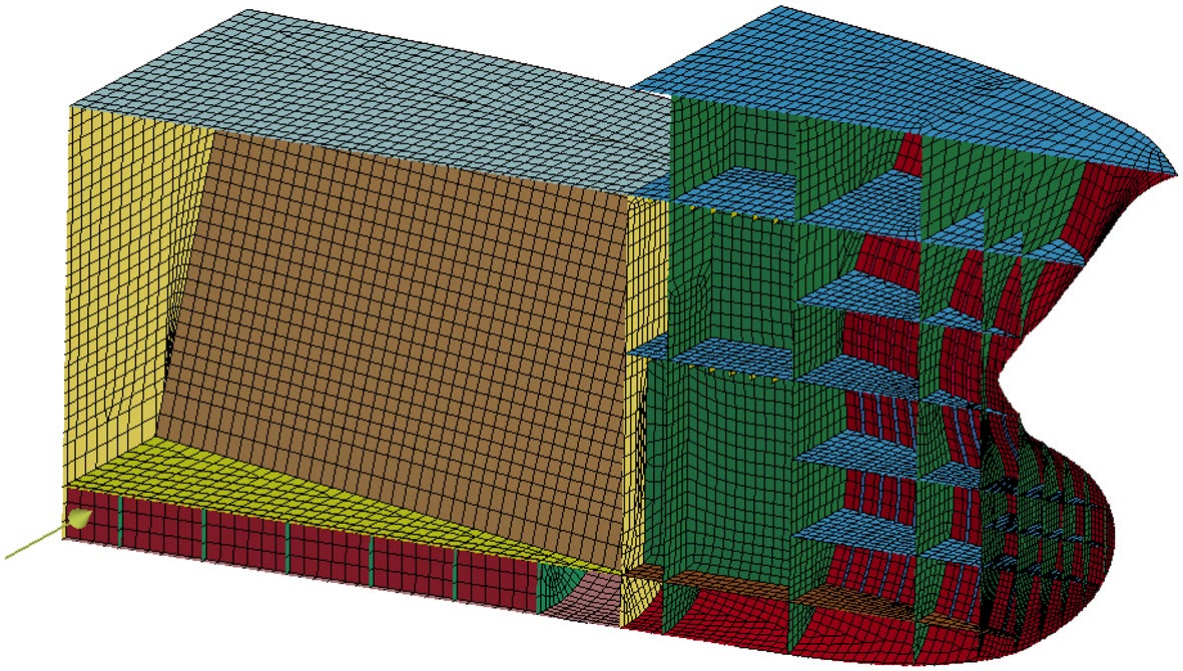
Bow FEM model, ANSYS/LS-DYNA.

This section presents some details on the FEM simulation of oil tanker bow forces. Commercial FEM software ANSYS/LS-DYNA^19,43^, was used to model the bow force pattern of a specific oil tanker in the Arctic Ocean. An explicit time-integration method is utilized by ANSYS LS-DYNA. As most nonlinear dynamics software employs explicit time-integration techniques, particularly when addressing ship-ice collision issues, this is a popular alternative. For non-iterative numerical methods, the explicit time-integration clearly outperforms the implicit time-integration in terms of CPU time savings. Both 2D and 3D analytic capabilities are included in the LS-DYNA program. It used the 3D LS-DYNA model, see^19,43^. The LS-DYNA code is commonly accepted in modern naval architecture. The ice-breaking model was built using the cellular automata theory, and three distinct ice-breaking regions with the identical density and thickness characteristics were built. Restart technology was unable to alter the positions of the components, thus the distance between them required to be determined and a finite element model created. The research object for this work is the bow of a double-bottom, double-hulled polar cargo ship. The bow's finite element model is seen in Fig. 4.

The bow of polar ships collides with the ice surface during the ice-breaking process, and the force of the collision is mostly focused around the waterline surface of the bow. This study ignores the ship's non-collision region and only takes into account the bow portion of the collision area in order to decrease calculation time and the computer memory occupation rate for structural calculations as well as the number of components in the entire model. The bow portion's hull construction matches that of the actual ship. For the hull model, the shell element with four free nodes is chosen. See Table 3 for the hull steel material parameters.

**Table 3:** Hull steel material parameters.

Name	Material parameter value
Hardening modulus ($E_{h}$)	$1.18 \times 10^{9} \;{\text{Pa}}$
Elastic Modulus ($E$)	$2.1 \times 10^{11} \;{\text{N}}/{\text{m}}^{2}$
Poisson's ratio ($\mu$)	0.3
Coefficient of viscosity (C)	40.5
Strain rate hardening parameter (P)	5

**Figure 5:**
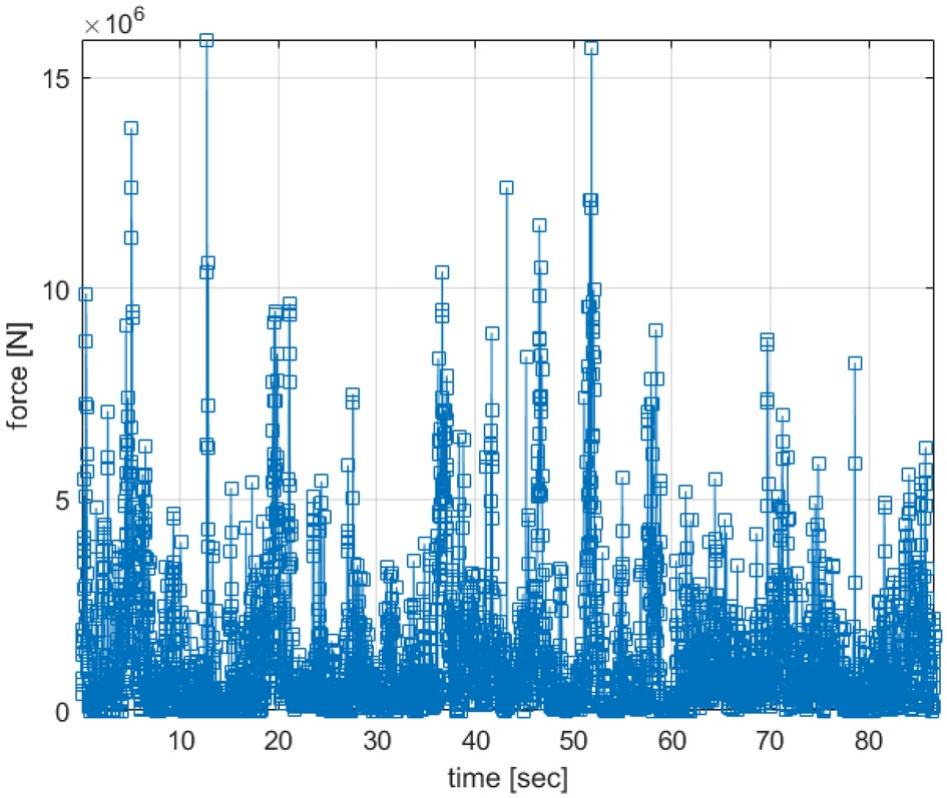
An example of total bow force for the broken ice model.

Figure 5 demonstrates a complete bow force example for the shattered ice model. The solid layer of the ice model was also simulated in addition to the shattered ice model only for comparison.

Therefore, the force acting on the tanker bow for the shattered ice model will be significantly less given the same ice qualities and vessel speed. According to IACS (The International Association of Classification Societies), PC4 polar boats have a bow force of around 16 NM^1^. The latter force value was found of the same order of magnitude as the bow force presented in Fig. 5. See also ISSC (International Ship and Offshore Structures Congress) committee report^24^.

From a structural point of view, the integrated bow load pattern is often more relevant to fatigue, while more detailed reliability study would require areal stress distribution along with structural deformation analysis. However, this study advocates general purpose reliability method, applicable not only to an integrated bow force, but any kind of stochastic process: either load or structural response type.

## Method

Let ${F}_{1},\dots ,{F}_{N}$ be consequent in time local maxima of the bow force process $F(t)$ at discrete monotonously increasing time instants ${t}_{1}<\dots <{t}_{N}$ within the target period $(0,T)$^9^. Bow force non-exceedance probability $P$ for the maximum force ${F}_{T}^{\mathrm{max}}=\underset{0\le t\le T}{\mathrm{max}}F\left(t\right)$

4 $$P =\mathrm{Prob}\{{F}_{T}^{\mathrm{max}}\le \eta \}$$

can be estimated as

$$P =\mathrm{Prob}\{{F}_{N}\le \eta ,\dots ,{F}_{1}\le \eta \}$$

$$=\mathrm{Prob}\left\{{F}_{N}\le \eta \right| {F}_{N-1}\le \eta ,\dots ,{F}_{1}\le \eta \}\cdot \mathrm{Prob}\{{F}_{N-1}\le \eta ,\dots ,{F}_{1}\le \eta \}$$

5 $$=\prod_{j=2}^{N}\mathrm{Prob}\{{F}_{j}\le \eta |{ F}_{j-1}\le \eta ,\dots ,{F}_{1}\le \eta \}\cdot \mathrm{Prob}({F}_{1}\le \eta )$$

In the following, the principle behind a cascade of approximations based on conditioning is outlined^11–21^. In practice, the dependence between neighbouring bow force maxima ${F}_{j}$ is not obviously negligible; thus, following one-step (will be called conditioning level $k=1$) memory approximation is introduced

6 $$\mathrm{Prob}\{{F}_{j}\le \eta |{ F}_{j-1}\le \eta ,\dots ,{F}_{1}\le \eta \}\approx \mathrm{Prob}\{{F}_{j}\le \eta |{ F}_{j-1}\le \eta \}$$

for $2\le j\le N$ (conditioning level $k=2$). The approximation introduced by Eq. (6) may be further expressed as

7 $$\mathrm{Prob}\{{F}_{j}\le \eta |{ F}_{j-1}\le \eta ,\dots ,{F}_{1}\le \eta \}\approx \mathrm{Prob}\{{F}_{j}\le \eta |{ F}_{j-1}\le \eta , {F}_{j-2}\le \eta \}$$

where $3\le j\le N$ (will be called conditioning level $k=3$), and so on. The idea is to monitor each independent failure that happened locally first in time, thus avoiding cascading local inter-correlated exceedances^10–15^. Equation (7) presents subsequent refinements of the statistical independence assumption. The latter type of approximations captures the statistical dependence effect between neighbouring maxima with increased accuracy^16–23^.

In the above, the stationarity assumption has been used. For non-stationary cases, an illustration may be as follows. Given the scattered diagram of $m=1,..,M$ sea states, each environmental short-term sea state has a probability ${q}_{m}$, so that $\sum_{m=1}^{M}{q}_{m}=1$. Next, let one introduce the long-term equation

8 $$P\equiv \sum_{m=1}^{M}P(m){q}_{m}$$

with $P(m)$ being the same function as in Eq. (2A) but corresponding to a specific short-term environmental sea state with the number $m$^44–50^.

## Results

**Figure 6:**
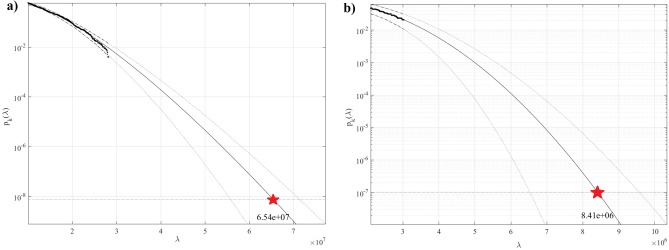
Extreme bow force predictions for (**a**) solid layer of ice model (left); (**b**) broken ice model (right). Dotted lines indicate extrapolated 95% confidence bands. Newtons on horizontal axis.

The statistical analysis findings for the severe bow force operating on the vessel during operations in ice conditions are presented in this section. For ease of use, the vessel's speed is set at a constant value of 2 m/sec. The distribution of ice thickness provided by Eq. (1) was utilised when simulating vessel bow dynamics with various ice thicknesses in ice conditions. Figure 6 presents modified Weibull method predicted 3 days extreme bow forces for solid layer of ice model, and for broken ice model, along with corresponding 95% confidence bands.

Since the original numerical simulation was just 90 s long, and extrapolation is typically done a few orders of magnitude down on the probability distribution decimal logarithmic scale, the authors have chosen 3 days return period purely for illustration. Bow force values with large return periods (about 3 days in this case, quite short, just for illustration) are essential for engineering design. Figure 6 presents extreme bow force predictions by extrapolated ${p}_{k}\equiv$ modified Weibull_k_ functions with conditioning level *k* = 6 for solid layer of ice model, and broken ice model. Dotted lines indicate the extrapolated 95% confidence interval (CI) bands. Conditioning level *k* was chosen according to the convergence of modified Weibull functions, see Sect. 5^7,51–59^.

Table 4 presents the maximal bow force predictions. The numerical maximal bow force predictions given in Table 4 are qualitative, as the oil tanker route was chosen purely exemplary, and the main focus of the current study was extreme value analysis methodology. Note that a similar approach can be applied to analyse extreme bow forces acting on any other ship model, as both numerical simulation approach as well as statistical model are of general design purpose.

**Table 4:** Bow force predictions for different ice models, 3-days return period.

Solid layer of ice model	Broken ice model
64.1 MN	7.8 MN

Regarding experimental validation of above-reported results: this study intends to draw research attention to the near future commercial Arctic navigation safety and reliability concerns, thus analyzed oil tanker model has not been neither approved, nor specifically designed yet. Model tests would require scaled vessel model, for example of the scale 1:50, but even then, apart from being expensive, – it is too premature to decide on specific vessel design. This is not an operation icebreaker, but only a hypothetical oil tanker of PC4.

## Conclusions

Safety and reliability are key issues for any vessel navigation and operation, especially in Arctic areas. This paper has studied collision forces exerted on the oil tanker bow. Two different ice loading models have been studied: a solid layer of ice model and a broken ice model with a random distribution of ice debris, simulated using the cellular automata model. The authors advocate the accurate yet straightforward reliability approach to estimate extreme loads, acting on the oil tanker bow in a realistic random ice loading environment. Since the onboard route-specific ice thickness data is often not available, there is a need for accurate and robust statistical methods at the design stage. The authors have applied the average conditional exceedance rate method to estimate extreme bow forces with a large return period. Predictions and proper confidence interval limits were given to indicate the practical merits of the suggested approach that could be easy for both onboard monitoring tools and engineers at the design stage. Note that a similar approach can be applied to analyse extreme bow forces.

This paper primarily focused on applying statistical techniques at the future vessel design stage. The bow force reported in this study was found in agreement with the one reported by IACS for the PC4 arctic vessel. The authors have performed a convergence study despite the absence of direct experiments.

## Data Availability

The datasets used and analyzed during the current study available from the corresponding author on reasonable request.
